# Comparing an optimised physiotherapy treatment package with usual physiotherapy care for people with tennis elbow — protocol for the OPTimisE pilot and feasibility randomised controlled trial

**DOI:** 10.1186/s40814-022-01132-x

**Published:** 2022-08-11

**Authors:** M. Bateman, B. Saunders, C. Littlewood, D. Davis, J. Beckhelling, K. Cooper, A. Skeggs, N. E. Foster, B. Vicenzino, J. C. Hill

**Affiliations:** 1 University Hospitals of Derby & Burton NHS Foundation Trust, Derby, UK; 2 School of Medicine, Keele University, Newcastle-under-Lyme, UK; 3 Faculty of Health, Social Care and Medicine, Edge Hill University, Ormskirk, UK; 4 Patient Representative, Derby, UK; 5 STARS Education and Research Alliance, The University of Queensland and Metro North Health, Herston, Australia; 6 School of Health & Rehabilitation Sciences: Physiotherapy, University of Queensland, St Lucia, Brisbane, Australia; 7 Derby Clinical Trials Support Unit , Royal Derby Hospital, Derby, UK

**Keywords:** Physiotherapy, Physical therapy, Lateral elbow tendinopathy, Tennis elbow, Pilot, Feasibility

## Abstract

**Background:**

Physiotherapy is recommended for people with tennis elbow, but whilst a wide array of treatments is available, the optimal approach remains uncertain. We have therefore recently developed an optimised physiotherapy treatment package for tennis elbow based on a synthesis of the evidence, patient input and clinical consensus. It consists of detailed advice and education, a structured progressive exercise programme and provision of a counter-force elbow brace. Here, we report the protocol for our multicentre pilot and feasibility randomised controlled trial (RCT) designed to (a) examine the feasibility of our optimised physiotherapy treatment package and (b) to pilot trial processes for a future fully powered RCT to test clinical and cost-effectiveness compared with usual physiotherapy treatment.

**Methods:**

A multicentre pilot and feasibility RCT will be conducted across three sites in England, recruiting up to 50 patients (or for a maximum of 12 months). Participants with tennis elbow, identified from physiotherapy clinic waiting lists and general practice surgeries, will be randomly allocated to receive the optimised physiotherapy treatment package or usual physiotherapy care. Analysis will focus on feasibility measures including consent rate, intervention fidelity, follow-up rate and outcome completion rate. A nested qualitative study will explore the acceptability of the study processes and patient and physiotherapist experiences of the new optimised intervention.

**Discussion:**

This study will determine the feasibility of a new optimised physiotherapy treatment package for people with tennis elbow and pilot the processes for a future fully powered RCT. In the longer term, this treatment package may provide superior clinical outcomes for patients, in terms of pain and quality of life, and be more cost-effective for the health service.

**Trial registration:**

Registered with the ISRCTN database 19/7/2021, https://www.isrctn.com/ISRCTN64444585

## Introduction

### Background and rationale

Tennis elbow, also known as lateral elbow tendinopathy, is a musculoskeletal condition that usually affects people in middle age and often affects an individual’s ability to work [1, 2]. In 2012, absenteeism from work due to tennis elbow cost the UK economy £27 M [3]. Common risk factors for onset have been identified as repetitive handling of heavy loads, forearm rotating motions, strong gripping force and working postures that combine force or load handling with raised arms [4]. Whilst the condition is self-limiting for many patients, 8.5–17% of individuals will have persistent symptoms, lasting more than 12 months [2, 5, 6]. Physiotherapy is recommended, but research has shown that physiotherapy treatment for tennis elbow varies widely and is suboptimal [7, 8]. In order to improve the physiotherapy treatment for people with tennis elbow, we recently developed an optimised physiotherapy treatment package by triangulating the best available research evidence, with consensus from UK physiotherapists with a special interest in tennis elbow, physiotherapy service managers and patient representatives, to ensure the intervention designed was acceptable to all stakeholders. A detailed description of this treatment package and how it was determined can be found in the open-access intervention development paper [9].

Improved physiotherapy provision for patients with tennis elbow has the potential to reduce pain, improve quality of life, reduce time off work and reduce the number of patients developing persistent symptoms. When physiotherapy fails to help, the usual pathway involves costly alternative treatments such as injections (of various types, e.g. autologous blood/platelet-rich plasma/corticosteroid/sodium hyaluronate) or surgery, but there is limited evidence for the effectiveness of these procedures [10, 11]. Surgery, however, is on the rise, for example in the USA there was a threefold increase between 2000 and 2011 [2]. In England, 2845 surgeries were performed in 2019/2020 at a cost of £1086 per procedure (total £3.1 million). If physiotherapy can be improved such that more patients with tennis elbow benefit, then fewer patients may subsequently require injections and surgery with associated cost-savings.

The newly designed optimised physiotherapy treatment package is therefore ready for feasibility and pilot trial testing, prior to conducting the main randomised controlled trial (RCT).

### Objectives

To report the protocol for a multicentre pilot and feasibility RCT, aimed at examining the feasibility of our optimised physiotherapy treatment package and piloting trial processes for a future fully powered RCT to test clinical and cost-effectiveness compared with usual physiotherapy treatment.

Specific objectives are as follows:

1. Estimate participant recruitment, outcome measure completion and follow-up rates in a pilot RCT.
2. Assess patient and clinician fidelity to the optimised intervention.
3. Explore the acceptability of the optimised physiotherapy treatment programme from both the perspective of patients and physiotherapists.

## Methods

### Trial design

**Fig. 1:**
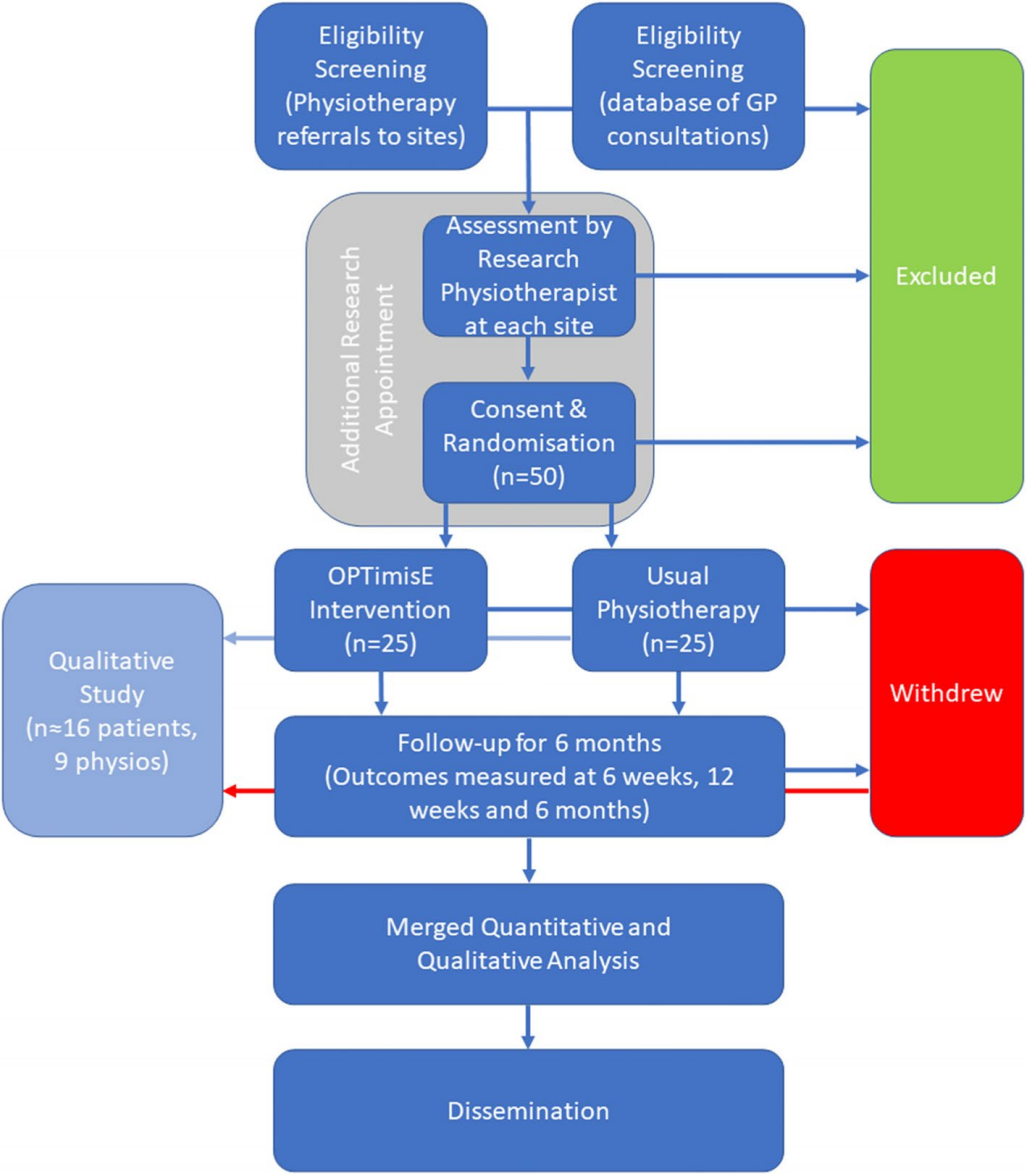
Study flowchart

This is a mixed-methods pilot and feasibility RCT with nested qualitative study (see Fig. 1).

### Study setting

The study will be conducted at three National Health Service (NHS) sites in England providing outpatient musculoskeletal physiotherapy for adult patients with tennis elbow.

### Patient eligibility criteria and identification

Patients will be included in the pilot and feasibility trial if they are adults aged 18 or over, with physiotherapist determined tennis elbow, have pain on palpation of the common extensor origin and on gripping and have either a positive Cozen’s, Mills', or Maudsley’s test [12]. Exclusion criteria include the following: a recent history of significant trauma to the affected limb, e.g. a fall on an outstretched hand, previous diagnosis of inflammatory arthritis or gout and previous diagnosis of osteoarthritis of the affected elbow, and neurological symptoms in the affected limb correlating with onset of elbow pain, e.g. loss of sensation in the hand, co-existing neck pain and stiffness that started at a similar time to the elbow symptoms, inability to understand English or lacking capacity for informed consent or are currently enrolled in another health-related research trial.

Patients with tennis elbow will be identified using one of two methods:

- By screening patient referrals at the three NHS primary care outpatient physiotherapy providers. Prior to attendance in the physiotherapy clinic, all primary care referrals will be screened by a physiotherapist, as is normal practice, and those patients who are potentially eligible will be sent a patient information sheet (PIS). The physiotherapist will then telephone the patient (typically 1–2 weeks later) to discuss the trial and book an appointment for an eligibility assessment with a research physiotherapist, if interested.
- By screening the SNOMED CT NHS database for patients in those three catchment areas with a diagnostic coding of tennis elbow in primary care within the last 3 months. Potentially eligible patients will be identified from the SNOMED CT database by a member of staff at participating GP practices in the locality. They will be sent a PIS by post along with a screening questionnaire and letter of introduction by the practice administrator. If interested and meeting the screening criteria, they will be asked to contact the Clinical Trials Unit (CTU) via the OPTimisE trial website. The principal investigator (PI) at the local trial site and their GP will then be informed of their interest to participate, and the GP will be requested to refer the patient to physiotherapy.

### Recruitment

Patients identified in the initial screening process will attend for clinical assessment by a physiotherapist trained in the OPTimisE protocol, to establish the diagnosis of tennis elbow and do a final confirmation of eligibility based upon the inclusion/exclusion criteria. Travel expenses for this initial patient visit (for eligibility assessment) will be offered. Patients meeting the eligibility criteria will be invited to participate in the RCT and consent gained as per Good Clinical Practice guidelines including an explanation of the condition, reassurance about receiving treatment, establishment of uncertainty as to the optimum physiotherapy treatment approach, an explanation of the study purpose, a balanced view of the two interventions, rights to withdraw, and an explanation of study procedures. There will be opportunity to discuss and ask questions before providing written consent via the trial consent form. Patients who decline to take part in the pilot and feasibility trial will be invited to be interviewed as part of the qualitative feasibility component. Those willing to be interviewed will be required to provide written consent to be contacted in relation to the interviews.

### Sample size

The pilot and feasibility RCT will recruit up to 50 participants or for a maximum of 12 months across three sites. These limits were a condition stipulated by the funder. The nested qualitative study will recruit until data saturation, estimated as up to 25 participants (approximately 16 patients and 9 treating physiotherapists).

### Assignment of interventions

#### Allocation sequence generation

Patients will be randomised in a 1:1 allocation (in mixed blocks) stratified by site using an online randomisation service provided by the CTU. Mixed blocks are required to reduce the predictability of the randomisation allocations.

#### Implementation and allocation concealment

The site PI or person delegated to take consent and randomise patients will use the online randomisation service ‘Sealed Envelope’ to access the allocation. This is an independent system to ensure allocation concealment.

#### Blinding

Due to the nature of physiotherapy treatments, it is not possible to blind participants to their treatment allocation. The treating physiotherapists will be blinded to the outcome measure data until the final results of the trial are reported. Outcome measure data will be collected and analysed by the research team.

### Interventions

Patients will be randomly allocated to receive either the optimised physiotherapy treatment package, by physiotherapists specifically trained to deliver this, or usual physiotherapy care delivered by other physiotherapists not trained in the optimised intervention but trained in the RCT procedures.

#### Usual care

Usual NHS physiotherapy will not be standardised in this pragmatic study, but the details of the content and number of treatments given will be captured at the end of a patient’s course of physiotherapy. The site PI, or delegated person at site, will review each patient’s physiotherapy notes and complete a case report form (CRF). Usual physiotherapy may involve a range of different treatments including advice and education, exercise, taping, manual therapy, acupuncture, ice therapy, orthotics, and massage [7, 8]. Evidence suggests that there is inconsistency of approach and a wide range of variation within treatment categories, for example the dosing of exercise therapy [8, 13].

#### Optimised intervention

The optimised physiotherapy treatment package includes patient advice and education, exercise therapy, and provision of a counterforce brace. It will differ from usual care by providing a detailed and consistent approach to treatment based upon best available evidence and omitting treatments lacking evidence of efficacy, such as taping, acupuncture and therapeutic ultrasound. The advice and education component is detailed and supported by high-quality written and videographic materials, developed in consultation with patients. The topics covered not only relate to tennis elbow but also incorporate modifiable lifestyle factors that may improve treatment response and reduce risk of recurrence (Table 1).

**Table 1:** The advice and education topics included in the optimised physiotherapy treatment package

Condition-specific advice	General/lifestyle factor advice
What tennis elbow is	Basic pain science
Activity modification	Promotion of self-efficacy
Pacing	General exercise advice
Ergonomics for work or sport	Smoking cessation (if applicable)
Medication advice	Sleep advice
	General diet advice
	Diabetes management (if applicable)

The exercise therapy component consists of a progressive regime incorporating stretching, isometric loading, concentric loading and eccentric loading designed to be adaptable to individual patient’s functional demands. Dosage is clearly defined based upon best evidence, and a novel aspect is that patients will be encouraged to exercise into levels of pain deemed acceptable by the individual patient. Painful exercise has been avoided in the interventions tested in the majority of tennis elbow trials to date, but recent systematic review evidence from the fields of back pain, shoulder pain and heel pain trials suggests it may offer improved short-term pain relief [14].

### Outcomes

The primary outcomes for this pilot and feasibility study are as follows (see Table 2):

- Consent rate
- Intervention fidelity in the intervention group
- Follow-up rate in the intervention group
- Outcome measure completion rate at 6 months

**Table 2:**
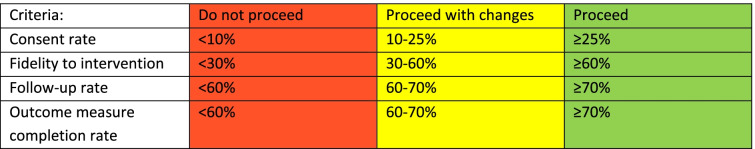
Feasibility criteria for a future main trial

The secondary outcomes for the study are as follows:

- Adherence to exercise therapy treatment
- Outcome measure completion rate at 6 weeks and 3 months
- Patient-reported outcomes (see Table 4)
- Completion of physical measures using the Squegg device
- Responsiveness analysis of outcome measures
- Acceptability of the optimised physiotherapy treatment package and trial processes, determined by the nested qualitative study

### Participant timeline (Table 3)

### Data collection methods

Once consented and prior to randomisation, participants will complete a baseline set of patient-reported outcome measures (PROMs) and demographic data including age, gender, ethnicity, duration of symptoms, occupation, education level, hand dominance and comorbidities. Physical measures of maximum grip strength and pain-free grip strength will be taken by the PI or person delegated to take consent, using an electronic grip strength measuring device (Squegg, https://mysquegg.com/). The Squegg is a US Food and Drug Administration (FDA)-approved dynamometer. Both measures will be repeated three times and a mean value used for analysis.

Patients will be offered the choice of two data collection methods for subsequent follow-up: (1) a paper questionnaire with follow-up questionnaires delivered and returned by post to the CTU or (2) an online system provided by Amplitude Clinical where the questionnaires are completed online using a smartphone, tablet or personal computer with automated follow-up questionnaire links delivered by email and SMS text message.

Patient-reported outcome follow-up data collection will be at 6 weeks, 3 and 6 months using the OPTimisE Follow-up Questionnaire. Patients who do not return the paper questionnaires within 2 weeks will receive reminder telephone calls and emails (according to patient preference) on up to two occasions from the chief investigator. Those who do not respond to the online questionnaires will receive automated reminders by email and SMS text message at 1 week and 2 weeks. The choice of outcome measures includes the core outcome set recently recommended for tennis elbow [21]. See Table 4 below:

The physical measures of maximum grip strength and pain-free grip strength will also be reported by patients using the OPTimisE Follow-up Questionnaire. Patients randomised to the OPTimisE intervention will be given a Squegg device to take home at their initial research visit with an information sheet on how to use it. The OPTimisE Follow-up Questionnaire will prompt the patients to use the Squegg device and document three measures each of maximum grip strength and pain-free grip strength. Patients in the usual physiotherapy group will be sent a Squegg device at 6 months. As the device can be used for grip strength training and is not provided as part of usual physiotherapy care, it will only be used at the 6-month time-point for patients in the usual physiotherapy group, to avoid intervention contamination.

Fidelity of the optimised physiotherapy intervention will be measured retrospectively by reviewing the CRF data to establish whether the treatments provided matched the pre-defined protocol. Fidelity will be calculated as a percentage based upon the number of applicable treatment items delivered. Any additional interventions will be noted and discussed in the final data analysis and report. Similarly, CRF data will be used to review the treatment of patients receiving usual physiotherapy to assess for key differences and similarities between the interventions and determine whether there is contamination between the interventions. Adherence to treatment will be measured in both treatment arms using a patient-reported exercise diary that is reviewed by the treating physiotherapist at each session and returned to the chief investigator by the patient after 3 months, via a stamped addressed envelope. Additionally, the 6-week, 3- and 6-month outcome questionnaires will include the Exercise Adherence Rating Scale [20]. Participants will receive a £20 voucher after completing all of the study questionnaires at 6 months.

### Nested qualitative study

#### Aims

The aim of the qualitative component of the feasibility assessment are to explore the following:

1. Patient views on the acceptability and suitability of the physiotherapy treatment received.
2. Patient views on study processes, e.g. experience of being recruited into the trial, reasons for declining participation, acceptability of study information and measurement of outcomes.
3. Clinician views on the acceptability and feasibility of delivering the optimised physiotherapy treatment package in an NHS setting.

#### Recruitment and sampling

Approximately, 16 participants from the pilot and feasibility RCT, including some that declined to take part, will be purposively sampled and interviewed by the chief investigator. Patients will be selected from both treatment arms and also from those that were eligible but did not consent to the trial but were willing to be interviewed. A varied sample will be obtained, in relation to characteristics such as trial site, age, gender, baseline tennis elbow pain and function and adherence to treatment (including patients that withdrew, if applicable).

Information relating to the qualitative interviews is included in the initial patient information sheet, and an option to give consent to further contact for the interviews is included in the consent form. Two months following initial assessment and randomisation, patients who have consented to further contact will be sent an invitation letter and patient information sheet before receiving a follow-up telephone call or email from the CI to confirm interest and arrange a suitable interview date. Patients will be able to decline participation in the interviews yet continue to be involved in the pilot and feasibility trial.

Similarly, approximately 9 physiotherapists involved with delivering the optimised physiotherapy intervention will be recruited from different sites. All physiotherapists on the trial delegation log will be approached with written information regarding the qualitative interviews, and from those that consent a broad sample of age, gender, clinical experience and site location will be sampled.

#### Qualitative data collection

Semi-structured interviews will be conducted with patient participants at least 2 months following randomisation either face to face, via video-conference or via telephone, depending on participant preferences. The interviews will be informed by topic guides developed in relation to the prespecified aims but also with PPIE input.

Physiotherapists will be interviewed following the end of their involvement with patients in the pilot and feasibility trial to allow time to reflect on their experiences of delivering the intervention to a number of patients in the RCT.

Consent will be obtained at the start of the interview, either in writing if the interview is face to face or audio recorded if the interview is over the telephone or video-conference, and checked again at the end. Interviews will be audio recorded and transcribed verbatim.

Participants that are interviewed will receive a £20 voucher for their time, and travel expenses will be reimbursed if the interviews are held in the physiotherapy department.

#### Qualitative data analysis

Anonymised interview transcripts will be analysed using thematic analysis, applying the COM-B model of behaviour change [22], to assess acceptability to recruitment and intervention delivery across the dataset in relation to views of patients and physiotherapists [23, 24]. Analysis will begin with the first data collection and continue until key theme data saturation is reached [25]. Using the theoretical framework of the COM-B model [21] allows us to more fully explore the aspects of patient and physiotherapist behaviour change [22]. The COM-B offers a way of understanding behaviour around three key determinants: *capability* — the psychological or physical ability to enact the behaviour, *opportunity* — the physical and social environment that enables the behaviour and *motivation* — the reflective and automatic mechanisms that activate or inhibit behaviour. The COM-B model is an extension of the earlier Theoretical Domains Framework (TDF) [26], which synthesises 112 psychological constructs determining behaviour change into 14 domains, which can be used to identify barriers and facilitators to behaviour change in the context of clinical interventions. The COM-B integrates these 14 domains within its three core components. The model has been successfully used in several recent studies exploring the feasibility of delivering complex interventions [27–29].

The following process will be adopted within and across both sets of participants, concurrent with iterative data collection to incorporate exploration/checking emerging themes and their implications in subsequent interviews:

1. Analysis will comprise distinct stages beginning with each case/transcript before moving to a cross-case comparison:

- *Level 1*: Read through individual transcripts/make preliminary notes.
- *Level 2*: Identify segments of meaning; apply initial codes.
- *Level 3*: Group initial labels to overarching within-case codes of meaning
- *Level 4*: Cross-case comparisons: looking for similarities, differences and inconsistencies, resulting in meta-codes spanning all cases/transcripts.
- *Level 5*: Cross data sets comparison, as above
- *Level 6*: Situating findings within the context of the physiotherapy interventions delivered and comparing responses between patients in the intervention group and the control group
- *Level 7*: Explore implications for intervention refinement for testing in a future main trial from the perspective of both the patient and the physiotherapist.

2. Analytic memos will be kept as the coding proceeds and will provide an audit trail to explain how codes develop and change.
3. A codebook consisting of a structured compendium of codes will be developed, including a description of how the codes are related to each other. It is anticipated that the codebook for physiotherapists and patients will differ in some respects.
4. Full coding team will analyse and agree codes and theoretical constructs at levels 3, 5, 6, and 7 and then map the themes to the three core components of capability, opportunity and motivation from the COM-B model.
5. The outcome will contribute to the assessment of future main trial feasibility and also provide valuable feedback that will be used to refine the intervention, supporting documentation and study processes.

### Data management

Data will be collected using a mix of paper and electronic methods. Where possible, a patient ID number will be used rather than identifiable information. Data from paper forms will be transcribed into an electronic database in Microsoft Excel stored on secure servers provided by the sponsor. Paper hard copies will be stored at the CTU and in the relevant Investigator Site Files. Study documentation will be stored securely (i.e. cupboards, shelves or filing cabinets with restricted access, e.g. within a locked office) to maintain participant confidentiality and study data integrity.

Electronic data captured at trial sites will be sent to the research team by secure NHS email. Online outcome data collection will be managed by Amplitude Clinical in ISO27001 Tier 3+ data centres approved for use by the NHS. Amplitude Clinical will not own the data — ownership is retained by the sponsor.

In order for patients to use the Squegg device, they will need to install the Squegg app on an Apple or Android smartphone/tablet. They are required to create an account within the app or log in using a Facebook or Google account. For data privacy reasons, participants will be recommended to use an email address rather than Facebook to create a user account; however, this is their own personal choice. No data will be shared between Squegg and the research team. Patients will use the device to measure grip strength and report the numerical values on the OPTimisE Follow-up Questionnaire.

Qualitative data will be organised and managed using NVivo software. Audio recordings and transcriptions will be stored on secure servers provided by the sponsor. An NHS-approved transcription service will be used that complies with data security regulations.

### Statistical methods

Descriptive statistics will be presented to summarise the distribution of baseline variables across each of the randomisation groups. The continuous baseline variables (e.g. age) will be reported with means, and 95% confidence intervals (95% CI), if shown to be normally distributed, using a combined skewness and kurtosis test, otherwise will be reported with medians and interquartile ranges (IQR). The categorical variables (e.g. gender) will be reported with frequencies and percentages.

A Consolidated Standards of Reporting Trials (CONSORT) flow diagram will be produced, showing the numbers and frequency of patients/participants: assessed for eligibility, reasons for ineligibility, screened as eligible, excluded before consent (and the frequency of each reason for exclusion), consented, excluded before randomisation (and the frequency of each reason for exclusion), randomised, allocated to each randomisation group, which received each allocated intervention, which did not receive each allocated intervention, lost to follow-up (and the frequency of each reason for loss to follow-up) for each analysis group, analysed for each analysis group and not analysed (and the frequency of each reason for not being analysed) for each analysis group.

Quantitative data analysis will be descriptive. This feasibility study aims to provide estimates of the consent, intervention fidelity, follow-up and outcome measure completion rates to inform a future main trial. By including 50 participants, we will be able to estimate a consent rate of 25% with a 95% confidence interval of (19%, 31%), an intervention fidelity of 60% with a 95% confidence interval of (46%, 74%) and a follow-up rate and outcome measure completion rate of 70% with a 95% confidence interval of (57%, 83%).

1. Consent rate — measured as a percentage of eligible patients approached to participate
2. Intervention fidelity in the intervention group — measured using CRF data. Fidelity to the intervention will be defined by delivery of at least 6 of the 12 prescribed advice/education topics, evidence of exercise prescription and progression in line with the protocol and provision of an elbow clasp splint. Results will be expressed as a percentage of the patients in the intervention group who received treatment assessed as being faithful to the protocol.
3. Follow-up rate in the intervention group — the actual number of visits (excluding the baseline visit) divided by the maximum number of possible visits
4. Outcome measure completion rate at 6 months — measured as a percentage of outcome measures completed across both the intervention and usual care groups

If any of the above feasibility outcomes are rated red (do not proceed) as per Table 2, then a future main trial will not be feasible. If any outcomes are rated amber (proceed with changes), but without any red, then a future main trial may be feasible with changes to the protocol. If all outcomes are rated green (proceed), then a main trial is feasible.

**Table 3:**
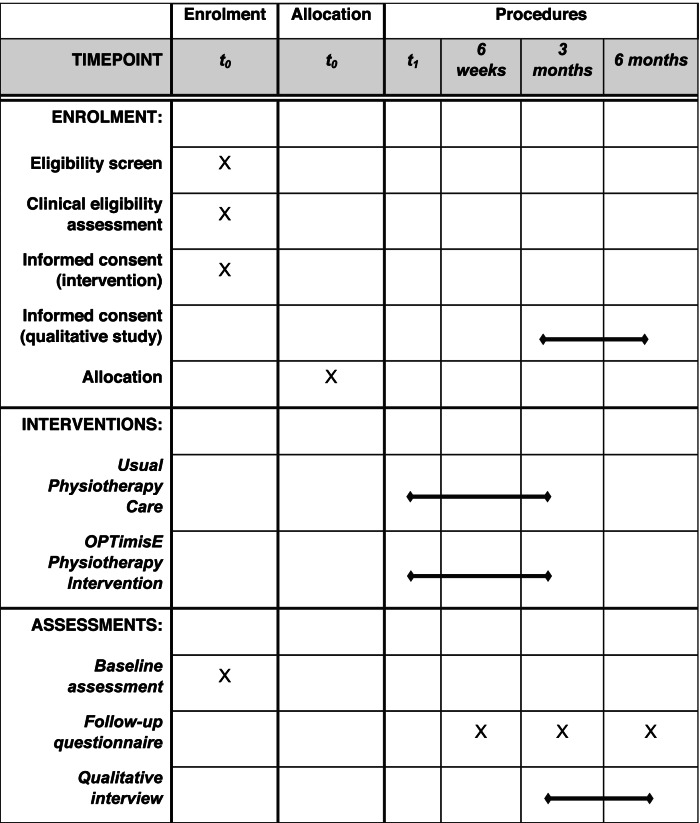
Schedule of events

*t*_0_ additional research clinic appointment *t*_1_ first physiotherapy treatment clinic appointment

Mean values and confidence intervals of the secondary outcomes (see Table 4) will be calculated if the data are normally distributed (this will be assessed using a combined test of skewness and kurtosis). If the data are not normally distributed, medians and interquartile ranges will be reported. The responsiveness of the different outcome measures will be compared, by calculating the treatment effect in the intervention group.

**Table 4:** Outcome measures and time-points

Outcome measure	Baseline	6 weeks	3 months	6 months
Numerical rating scale of pain on gripping	X	X	X	X
Patient-rated tennis elbow evaluation (PRTEE) [15]	X	X	X	X
Tampa scale of kinesiophobia (TSK-11) [16]	X	X	X	X
Pain self-efficacy questionnaire (PSEQ) [17]	X	X	X	X
EQ 5D5L [18]	X	X	X	X
Pain-free grip strength (lbs)	OPTimisE group X	OPTimisE group X	OPTimisE group X	OPTimisE group X
	Usual care group X	Usual care group	Usual care group	Usual care group X
Maximum grip strength (lbs)	OPTimisE group X	OPTimisE group X	OPTimisE group X	OPTimisE group X
	Usual care group X	Usual care group	Usual care group	Usual care group X
Global perceived effect (GPE-11) [19]		X	X	X
Exercise adherence rating scale (EARS) [20]		X	X	X

### Data monitoring and auditing

The site PIs must ensure that source documents and other documentation for this study are made available to study monitors, the REC or regulatory authority inspectors. Authorised representatives of the sponsor will visit the participating sites on two occasions to conduct audits/inspections.

### Harms

All adverse events (AEs) and serious adverse events (SAEs) will be recorded and reviewed from the time of written informed consent until 6 months following the first intervention.

All AEs and SAEs occurring during the duration of the study must be recorded by the site PI and sent to the CI within 48 h for review.

All related and unexpected SAEs must be reported by the CI using the ‘non-CTIMP safety report to REC form’ from the HRA website. The completed form should be submitted to the sponsor and REC within 15 days of the CI becoming aware of the event. Safety information will be reviewed during trial management group meetings.

## Discussion

This study will determine the feasibility of a new optimised physiotherapy treatment package for people with tennis elbow and pilot the processes for a future fully powered RCT. In the longer term, this treatment package may provide superior clinical outcomes for patients, in terms of pain and quality of life, and be more cost-effective for the health service.

The study is designed to be pragmatic and deliverable in a primary care setting. For this reason, the inclusion/exclusion criteria require no additional imaging or specialist assessment, and the usual care arm has not been standardised. The components of usual care and the OPTimisE treatment protocol that are delivered in practice will be documented as described in the ‘Data collection methods’ section. Subsequently, one of the feasibility outcomes is to assess the content of the usual care treatments given, compared to the OPTimisE treatment, to decide whether they are sufficiently different.

The optimised intervention design was based upon the principles underpinning the development of complex healthcare interventions [30], combining the best available evidence (acknowledging that this may not have all been high quality due to the limitations of the evidence base) and the input of relevant stakeholders. These included expert clinicians, service managers and patients who had experienced tennis elbow. One of the greatest limitations of the evidence base related to physiotherapy for this patient population is the lack of studies with follow-up over 3 months. Given the importance of longer-term follow-up in this persistent and/or recurrent musculoskeletal condition, it is important that we are confident that longer-term follow-up is feasible, hence the selection of the 6-month time-point as a primary outcome.

## Data Availability

Data sharing is not applicable to this article as no datasets were generated or analysed during the current study. Materials will not be made available until the completion of a future main trial, if applicable.

## Abbreviations

AE: Adverse event
CI: Chief investigator
CRF: Case report form
GCP: Good clinical practice
ISF: Investigator site file
ISRCTN: International Standard Randomised Controlled Trial Number
NHS: National Health Service
PI: Principal investigator
PIS: Participant information sheet
PPIE: Patient and public involvement and engagement
RCT: Randomised controlled trial
REC: Research ethics committee
SAE: Serious adverse event
